# Right ventricular global constructive work as an echocardiographic predictor of worsening heart failure

**DOI:** 10.1093/ehjopen/oeag019

**Published:** 2026-02-17

**Authors:** Aram Chilingaryan, Lusine Tunyan, Hamlet Hayrapetyan, Milena Arzumanyan, Hovik Balyan

**Affiliations:** Yerevan State Medical University Named After Mkhitar Heratsi, 2 Koryun St, Yerevan 0025, Armenia; Yerevan Scientific Medical Center, 7 Nersisyan St, Yerevan 0014, Armenia; Yerevan State Medical University Named After Mkhitar Heratsi, 2 Koryun St, Yerevan 0025, Armenia; Yerevan Scientific Medical Center, 7 Nersisyan St, Yerevan 0014, Armenia; Yerevan State Medical University Named After Mkhitar Heratsi, 2 Koryun St, Yerevan 0025, Armenia; Yerevan Scientific Medical Center, 7 Nersisyan St, Yerevan 0014, Armenia; Yerevan Scientific Medical Center, 7 Nersisyan St, Yerevan 0014, Armenia

**Keywords:** Right ventricular function, Echocardiographic myocardial work, Pressure–strain loop, Heart failure, Worsening heart failure, Pulmonary pressures

## Abstract

**Aims:**

Worsening heart failure (WHF) is a pivotal event in the trajectory of chronic heart failure, yet early risk stratification remains challenging. Echocardiographic right ventricular myocardial work (RVMW), a pressure–strain–integrated index, may offer incremental prognostic value beyond conventional echocardiographic and biomarker-based markers. This study assessed whether right ventricular global constructive work (RVGCW), the principal component of RVMW, predicts WHF and cardiovascular mortality.

**Methods and results:**

In this prospective study, we enrolled 215 ambulatory patients with chronic heart failure New York Heart Association Functional Class (II–IV), encompassing heart failure with reduced ejection fraction, heart failure with mildly reduced ejection fraction, and heart failure with preserved ejection fraction, all in sinus rhythm and receiving guideline-directed medical therapy. Comprehensive transthoracic echocardiography was performed using a GE Vivid E95 system with offline analysis (EchoPAC v204). Right ventricular myocardial work was quantified using a non-invasive pressure–strain loop methodology, and RVGCW was prespecified as the primary RVMW parameter of interest. Right ventricular global constructive work was derived from right ventricular pressure–strain loops integrating right ventricular (RV) global strain and estimated pulmonary pressures. Patients were followed for 24 months with scheduled 3-month assessments and additional visits for suspected WHF. The primary endpoint was composite WHF, defined as heart failure hospitalization or adjudicated subclinical WHF.

Baseline RVGCW was significantly lower in patients who developed WHF compared with those who did not (607 ± 48 vs. 648 ± 52 mmHg%, *P* < 0.001). In multivariable Cox models adjusting for N-terminal pro-B-type natriuretic peptide, left atrial reservoir strain, left ventricular global longitudinal strain, left atrial volume index, tricuspid annular plane systolic excursion/systolic pulmonary artery pressure, and RV strain, RVGCW remained independently associated with WHF (hazard ratio 0.978, 95% confidence interval 0.973–0.998, *P* = 0.014). In receiver operating characteristic analysis, a cohort-derived RVGCW threshold demonstrated high discrimination for WHF (area under the curve 0.90).

**Conclusion:**

Echocardiographic RVGCW is a strong, independent predictor of WHF and provides substantial incremental prognostic information beyond traditional markers; its association with cardiovascular mortality was exploratory.

## Graphical Abstract

**Graphical Abstract:**
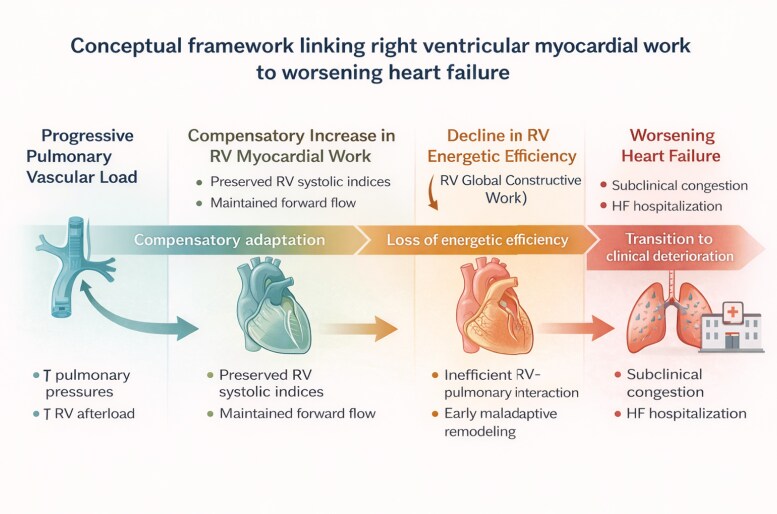

## Introduction

Chronic heart failure (HF) remains a major global health burden, with high rates of morbidity, mortality, and healthcare resource utilization.^1^ Its prevalence continues to rise across both high- and middle-income countries.^2,3^ Despite advances in guideline-directed medical therapy (GDMT), a significant proportion of patients experience worsening heart failure (WHF), which is considered as a distinct and critical phase in the natural history of HF that signals disease progression and portends poor outcomes.^1,4^

The onset of WHF is often insidious. Early signs may be asymptomatic or masked by reduced physical activity, particularly in the outpatient setting, leading to delayed recognition and suboptimal treatment.^4^ Hospitalization for WHF is associated with increased rates of rehospitalization and mortality, irrespective of left ventricular (LV) ejection fraction (EF).^1^ Early identification of patients at risk is therefore crucial for optimizing management and improving prognosis.

Current tools for WHF prediction remain limited. Biomarkers such as N-terminal pro-B-type natriuretic peptide (NT-proBNP) and troponin can detect subclinical decompensation,^5–7^ but their levels may vary significantly without a clear correlation to filling pressures, and biomarker-guided strategies have not consistently improved outcomes compared with standard care.^8^ Clinical predictors, including weight gain, increased dyspnoea, and B-lines on lung ultrasound, often manifest shortly before overt decompensation and may be missed if patients lack timely access to medical care. Other clinical variables (age, hypotension, tachycardia, or worsening renal function) may not be present early in the trajectory.

Although several risk scores have been developed to estimate WHF or mortality risk in chronic HF,^9,10^ no widely validated models exist for patients with recent WHF episodes. Similarly, the role of non-invasive telemonitoring remains uncertain. While some studies demonstrate benefit,^11,12^ others have shown neutral results, and current international guidelines do not recommend its routine use.^13,14^

There is therefore a critical unmet need for reliable, measurable, and persistent predictors of WHF, ideally capable of identifying patients in the preclinical or early phase of decompensation.

Right ventricular (RV) dysfunction is often associated with more advanced HF and worse outcomes. Because RV performance reflects both intrinsic myocardial contractility and afterload, right ventricular myocardial work (RVMW), a parameter integrating pressure and deformation, may provide incremental prognostic value beyond conventional metrics.

We conducted this study to evaluate the prognostic role of RVMW in chronic HF. While previous studies have demonstrated the feasibility of RVMW assessment and its association with invasive haemodynamics or RV dysfunction in selected cohorts, no study has examined RVMW as a longitudinal predictor of WHF in ambulatory patients across HF phenotypes.

Specifically, we aimed to determine whether right ventricular global constructive work (RVGCW), an echocardiographic pressure–strain–integrated marker, provides incremental prognostic value beyond established echocardiographic indices and biomarkers, independent of LVEF.

## Methods

### Study population

A total of 215 patients with chronic HF classified as New York Heart Association functional Class II–IV (48% female, mean age 58 ± 7 years) were prospectively enrolled. All participants were in sinus rhythm and receiving GDMT and had experienced at least one hospitalization within the previous 6 months. Patients with heart failure with reduced ejection fraction (HFrEF), heart failure with mildly reduced ejection fraction (HFmrEF), and heart failure with preserved ejection fraction (HFpEF) were evenly represented. All patients were clinically stable and appropriately decongested before enrolment.

The primary endpoint was composite WHF, defined as either hospitalization for HF or adjudicated subclinical WHF. All patients underwent echocardiographic examination with good acoustic windows, and only those with well-defined tricuspid regurgitation (TR) and pulmonary regurgitation (PR) envelopes were included. Patients were followed for 24 months and assessed every 3 months with clinical evaluation, NT-proBNP testing, and focused echocardiography to detect subclinical WHF at an early stage. Additional unscheduled visits were performed if patients developed symptoms suggestive of WHF.

Patient selection was performed prospectively from consecutive ambulatory patients referred for chronic HF follow-up. Of all patients screened, those with atrial fibrillation, inadequate acoustic windows, or non-analysable TR Doppler envelopes were excluded. The final study cohort consisted of 215 patients. Patient selection, exclusions, and final inclusion are summarized in Supplementary material online, *Figure S1*.

### Comorbidities and clinical definitions

Worsening heart failure was classified as clinical or subclinical. Subclinical WHF was defined by the presence of at least two objective criteria in the absence of overt symptoms, including (i) new or increased pulmonary congestion assessed by lung ultrasound B-lines, (ii) dilated and poorly collapsible inferior vena cava, and/or (3) a ≥30% increase in NT-proBNP from baseline.

All WHF events were adjudicated by two independent cardiologists blinded to myocardial work results. In cases of disagreement, events were resolved by consensus. Lung ultrasound and inferior vena cava assessments were performed using standardized protocols.

We acknowledge that subclinical WHF definitions are not yet universally standardized across centres; however, a structured adjudication process was used to minimize operator dependence.

Hyperlipidaemia was defined according to the patient’s serum lipid profile at baseline. Diabetes mellitus (DM) was defined by a fasting plasma glucose level of ≥126 mg/dL or use of oral hypoglycaemic agents or insulin. Chronic obstructive pulmonary disease was identified based on documented prior diagnosis in the medical record. Arterial hypertension (AH) was diagnosed as an office blood pressure of ≥140 mm Hg systolic and/or ≥90 mm Hg diastolic, confirmed by three consecutive measurements according to standard recommendations, or by a documented prior history of AH. Prior myocardial infarction (MI) was determined from medical records. Coronary artery disease (CAD) was defined as a documented history of MI, percutaneous coronary intervention (PCI), or coronary artery bypass grafting (CABG).

Anaemia was defined as a haemoglobin concentration of <13 g/dL in men and <12 g/dL in women. Glomerular filtration rate (GFR) was estimated using a standard estimation equation.^15^ Body mass index (BMI) was calculated from height and weight measured at enrolment. Comorbidity burden was assessed using the Charlson comorbidity index.^16^ Information on baseline medical therapy, including β-blockers, angiotensin-converting enzyme inhibitors (ACE-Is), angiotensin receptor blockers (ARBs), and mineralocorticoid receptor antagonists (MRAs), was obtained from medical records.

### Echocardiography

Comprehensive two-dimensional (2D) and three-dimensional (3D) transthoracic echocardiography was performed by a single experienced echocardiographer using GE Vivid E95 (software version 204) with an M5SC-D probe with a frequency of 1.7–3.3 MHz. Three consecutive cardiac cycles were recorded. Two-dimensional acquisitions were obtained at 50–90 frames/s, while 3D LVQ and RVQ analyses were performed at a temporal resolution of ∼60 frames/s. All linear and volumetric measurements, as well as the assessment of LV filling pressures, were performed according to joint recommendations of the American Society of Echocardiography and the European Association of Cardiovascular Imaging.^17,18^

Three-dimensional datasets were acquired using full-volume acquisition over six cardiac cycles during breath-hold at optimal image quality.

All images were stored and analysed offline by two independent investigators blinded to the study objectives, using EchoPAC v204 (General Electric Vingmed Ultrasound, USA). Primary analyses were performed by a single experienced observer; reproducibility analyses were conducted as described below.

Left ventricular ejection fraction with area strain (AS) and right ventricular ejection fraction (RVEF) were measured using 3D LVQ and 3D RVQ analysis, respectively.

Left ventricular filling pressures were considered elevated if ≥2 of the following criteria were present, in accordance with American Society of Echocardiography/European Association of Cardiovascular Imaging guidelines: average E/e′ > 14, left atrial volume index (LAVI) > 34 mL/m², and TR peak velocity > 2.8 m/s.^18^ In patients with intermediate or inconclusive results, left atrial reservoir strain (LASr) ≤ 18% was used as an additional criterion to support the presence of elevated LV filling pressures.^19^ If findings remained inconclusive, a diastolic stress test was performed in accordance with current recommendations.^18^

Speckle-tracking strain analysis was performed offline with Q-analysis on EchoPAC. Endocardial borders were manually traced in three apical views after which the software automatically delineated myocardial walls. Manual adjustments were made when necessary. Global longitudinal strain (GLS) values were derived automatically and reported as absolute values.

Left ventricular myocardial work (LVMW) parameters were calculated from non-invasive pressure–strain loops, obtained by integrating 2D speckle-tracking strain with brachial cuff blood pressure measured in the left lateral decubitus position after 5 min of rest. Valvular events timing was defined using transmitral and aortic Doppler flows.

Left ventricular global work index (LVGWI) was calculated as the total area within the pressure–strain loop. Left ventricular global constructive work (LVGCW) represented the sum of positive work during systolic shortening and negative work during isovolumic relaxation.

Left ventricular global wasted work (LVGWW) represented energy loss during myocardial lengthening in systole and shortening during isovolumic relaxation. Left ventricular global work efficiency (LVGWE) was calculated as the ratio of constructive work to the sum of constructive and wasted work, expressed as a percentage.

Left atrial reservoir strain and LAVI were measured using AFI LA software on EchoPAC.

Right ventricular strain in RV-focused apical four-chamber view was assessed using AFI RV on EchoPAC and reported as absolute values. For RVMW calculations, RV global strain was obtained using Q-analysis software analogous to LV strain analysis. Event timings were determined using Doppler valve dynamics. Pulmonary valve opening and closure were identified from the onset and termination of the pulmonary outflow Doppler envelope. Tricuspid valve opening and closure were defined by the onset and end of transtricuspid inflow on pulsed-wave Doppler. Right ventricular myocardial work was derived from RV strain in the RV-focused apical four-chamber view, measured three times to generate a complete MW bull’s-eye plot.^20^

Pulmonary artery pressures were estimated non-invasively. Systolic pulmonary artery pressure (sPAP) was calculated as the sum of the maximal transtricuspid pressure gradient (PGmax) derived from TR peak velocity and estimated right atrial pressure (RAP) based on @IVC diameter and collapsibility. Mean pulmonary artery pressure (mPAP) was derived from PGmean between the RV and RA plus RAP. Diastolic pulmonary artery pressure (dPAP) was calculated as dPAP = 1.5 × [mPAP − (sPAP/3)]. Valvular opening and closure timing of pulmonic valve was set with pulmonary systolic Doppler flow and tricuspid opening and closure timings were set from direct visualization of the valve leaflets on an RV-focused apical four-chamber view.

Using RV pressure–strain loop analysis, the following RVMW components were derived: right ventricular global work index (RVGWI), RVGCW, right ventricular global wasted work (RVGWW), and right ventricular global work efficiency (RVGWE). A graphical illustration of RVMW assessment is provided in the *Graphical abstract* and Supplementary material online, *Figure S2*. Right ventricular global constructive work was prespecified as the primary RVMW parameter for all prognostic analyses, while other indices were reported for mechanistic and descriptive purposes.

Tricuspid annular plane systolic excursion/sPAP ratio was measured in all patients as an additional marker of RV–pulmonary coupling.^21^

All patients were also scanned for the presence of B-lines on lung ultrasound.

### Statistical analysis

Continuous variables are presented as mean ± standard deviation or median interquartile range and categorical variables as frequencies and percentages. Group comparisons were performed using the Student’s *t*-test or Mann–Whitney *U* test for continuous variables and the χ² or Fisher’s exact test for categorical variables, as appropriate.

The prognostic value of echocardiographic parameters was evaluated using receiver operating characteristic (ROC) curve analysis, with the area under the curve (AUC) and optimal cut-off values determined using the Youden index.

Event-free survival was analysed using the Kaplan–Meier method, and differences between survival curves were assessed with the log-rank test.

In the primary analysis, patients were censored at the time of the first WHF event. For secondary analyses focusing specifically on HF hospitalization, patients with preceding subclinical WHF were not censored and remained at risk until hospitalization, death, or end of follow-up. Secondary analyses were performed separately for subclinical WHF and HF hospitalization to assess the consistency of RVGCW prognostic performance across event types.

Univariable and multivariable Cox proportional hazards regression models were used to identify predictors of the primary outcome, and results are reported as hazard ratios (HRs) with 95% confidence intervals (CIs). The incremental prognostic value of RVGCW was assessed using nested Cox models and likelihood ratio testing.

For intra-observer reproducibility, the primary investigator re-analysed 25 randomly selected image datasets 2 weeks after the initial assessment, blinded to the original measurements. For inter-observer reproducibility, a second independent investigator, blinded to the results of the first, analysed the same 25 datasets. Intraclass correlation coefficients (ICCs) with 95% CI were calculated using a two-way random-effects model with absolute agreement. Intraclass correlation coefficient values were interpreted as <0.50 poor, 0.50–0.75 moderate, 0.75–0.90 good, and >0.90 excellent agreement.

A two-sided *P* < 0.05 was considered statistically significant. To reduce the risk of model overfitting, multivariable Cox regression models were deliberately limited to clinically relevant and non-redundant variables. Sensitivity analyses were performed using reduced models, sequentially excluding correlated RV parameters (e.g. RV strain or TAPSE/sPAP). The association between RVGCW and WHF remained consistent across all sensitivity models.

Statistical analyses were performed using SPSS v25.0 (IBM Corp., Armonk, NY, USA) and R v4.3.1 (R Foundation for Statistical Computing, Vienna, Austria).

## Results

A total of 215 patients with chronic HF were followed for a median of 24 months. Seventy-three patients (34%) experienced WHF, and 14 patients (6.5%) died from cardiovascular causes. Baseline demographic and echocardiographic characteristics across HF phenotypes (HFrEF, HFmrEF, and HFpEF) are shown in *Tables 1* and *2*. Secondary echocardiographic variables not central to the primary hypothesis were moved to supplementary tables to improve clarity and focus of the main manuscript.

**Table 1:** Baseline clinical and demographic characteristics across heart failure phenotypes

Variable	HFrEF	HFmrEF	HFpEF
*n* (%)	72 (33.5)	70 (32.5)	73 (34.0)
Gender male *n* (%)	54 (75.0)	48 (68.6)	21 (28.8)*
Age yr	59 ± 8	63 ± 7	74 ± 9*
Smokers *n* (%)	58 (80.5)*	41 (58.6)	28 (38.4)
CAD *n* (%)	53 (73.6)*	43 (61.4)	32 (43.8)
AH *n* (%)	57 (79.2)	42 (64.3)	68 (93.1)*
DM *n* (%)	23 (31.9)	28 (40.0)	39 (53.4)*
Anaemia *n* (%)	9 (12.5)	13 (18.6)	19 (26.0)*
CKD *n* (%)	8 (11.1)	9 (12.9)	27 (37.0)*
Charlson CI > 3 *n* (%)	5 (6.9)	9 (12.9)	21 (28.8)*
Chronic obstructive pulmonary disease *n* (%)	11 (15.3)	13 (18.6)	25 (34.2)*
NT-pro BNP (pg/mL)	1849 ± 158	1756 ± 167	982 ± 118*
ACE/ARB/ARNI *n* (%)	70 (97.2)	68 (97.1)	51 (69.9)*
B-blockers *n* (%)	71 (98.6)	68 (97.1)	32 (43.8)*
Spironolactone *n* (%)	65 (86.7)	63 (90.0)	44 (60.3)*
SGLT2I *n* (%)	68 (94.4)	69 (98.5)	69 (94.5)

Values are presented as mean ± standard deviation or number (%).

Between-group comparisons were performed using one-way analysis of variance (ANOVA) for continuous variables and χ² test for categorical variables.

HFrEF, heart failure with reduced ejection fraction; HFmrEF, heart failure with mildly reduced ejection fraction; HFpEF, heart failure with preserved ejection fraction; CAD, coronary artery disease; AH, arterial hypertension; DM, diabetes mellitus; CKD, chronic kidney disease; NT-proBNP, N-terminal pro-B-type natriuretic peptide. * *P* < 0.01 compared with the corresponding parameters of both other groups.

**Table 2:** Echocardiographic characteristics across heart failure phenotypes

	HFrEF	HFmrEF	HFpEF
LV GLS (%)	12.2 ± 3,1	14.2 ± 1.8	17.1 ± 3.6*
E/e′avg	15.3 ± 5.1	13.2 ± 4.2*	16.4 ± 2.3
LAVI (mL/m^2^)	32.1 ± 8.3	29.8 ± 7.2	38.0 ± 9.8*
LASr (%)	31.8 ± 10.9	29.4 ± 12.8	15 ± 9.3*
TR PGmax (mmHg)	40.7 ± 20.2	38.4 ± 15.6^&^	44.7 ± 15.8*
TAPSE/sPAP	0.57 ± 0.12	0.61 ± 0.14	0.57 ± 0.15
RV GS (%)	20.8 ± 2.6	21.3 ± 2.9	20.2 ± 3.4
RV GWI (mmHg%)	801 ± 23	785 ± 22	829 ± 31*
RV GCW (mmHg%)	644 ± 32	632 ± 23	641 ± 29
RV GWW (%)	119 ± 12	121 ± 14	122 ± 11
RV GWE (mmHg%)	84 ± 5	85 ± 7	84 ± 9

Values are presented as mean ± standard deviation.

Between-group comparisons were performed using one-way ANOVA with Bonferroni *post hoc* correction.

LV, left ventricle; RV, right ventricle; GLS, global longitudinal strain; GWI, global work index; GCW, global constructive work; GWW, global wasted work; GWE, global work efficiency; LASr, left atrial reservoir strain; LAVI, left atrial volume index; TAPSE, tricuspid annular plane systolic excursion; sPAP, systolic pulmonary artery pressure.

^*^ *P* < 0.01 compared with the corresponding parameters of both other groups.

Patients with HFrEF were younger, mostly men, and smokers with CAD and AH with fewer comorbidities. Patients with HFpEF were older and mostly women with AH, DM, anaemia, chronic obstructive pulmonary disease, or CKD. Though HFpEF patients had less prevalence of CAD compared with HFrEF patients, still prevalence of CAD was high (43.8%).

Patients’ EchoCG data are shown in *Table 2*.

### Reproducibility

Intra- and inter-observer reproducibility for RV myocardial work indices was excellent, with ICCs ranging from 0.939 to 0.984 (*P* < 0.01) and 0.912 to 0.972 (*P* < 0.01), respectively (*Table 3*).

**Table 3:** Intra- and inter-observer reproducibility of right ventricular myocardial work parameters

Variable	Intra-observer agreement ICC (95% CI)	Inter-observer agreement ICC (95% CI)
RV GS	0.979 (0.947–0.992)*	0.967 (0.937–0.981)*
RVGWI	0.984 (0.958–0.996)*	0.972 (0.948–0.989)*
RVGCW	0.951 (0.934–0.973)*	0.956 (0.931–0.968)*
RVGWW	0.968 (0.939–0.981)*	0.912 (0.887–0.938)*
RVGWE	0.939 (0.884–0.956) *	0.948 (0.923–0.964)*

Agreement was assessed using intraclass correlation coefficients calculated with a two-way random-effects model and absolute agreement. RVGS, right ventricular global strain; RVGWI, right ventricular global work index; RVGCW, right ventricular global constructive work; RVGWW, right ventricular global wasted work; RVGWE, right ventricular global work efficiency.

^*^ *P* < 0.01 for all intraclass correlation coefficient values.

### Worsening heart failure

Patients who developed WHF exhibited significantly higher LV filling pressures, impaired LA reservoir strain and increased LA volume, higher NT-proBNP levels, and more pronounced RV dysfunction at the time of WHF detection (*Tables 4–5*).

**Table 4:** Comparison of echocardiographic variables in patients with and without worsening heart failure within each heart failure phenotype

WHF *n* (%)	HFrEF		HFmrEF		HFpEF	
	28 (34)		26 (32)		28 (34)	
Variable	No WHF	WHF	No WHF	WHF	No WHF	WHF
E/e′avg	15.3 ± 5.1	18.0 ± 6.2*	13.5 ± 4.7	16.4 ± 5.2*	15.9 ± 2.1	19.8 ± 3.7*
LAVI (mL/m^2^)	32.9 ± 7,8	33.8 ± 8.1	28.6 ± 8.9	32.1 ± 9.2	38.3 ± 9.1	39.1 ± 9.8
LASr (%)	29.1 ± 9.4	23.0 ± 7.8*	27.8 ± 13.2	21.7 ± 10.6*	19.0 ± 6.7	14 ± 5.3*
TAPSE/sPAP	0.59 ± 0.14	0.49 ± 0.13*	0.62 ± 0.12	0.52 ± 0.18	0.58 ± 0.16	0.51 ± 0.11*
RV GS (%)	22.1 ± 2.9	18.4 ± 3.8*	21.9 ± 3.2	17.9 ± 4.1*	20.3 ± 4.1	19.8 ± 3.1*
RV GWI (mmHg%)	875 ± 27	810 ± 31*	781 ± 29	752 ± 32*	820 ± 42	918 ± 51
RV GCW (mmHg%)	651 ± 39	618 ± 42**	649 ± 34	623 ± 41*	645 ± 34	619 ± 28**
RV GWW (%)	110 ± 18	125 ± 21*	112 ± 21	128 ± 29	110 ± 14	135 ± 18
RV GWE (mmHg%)	87 ± 5	83 ± 5*	85 ± 9	83 ± 7	86 ± 9	82 ± 7*

Values are presented as mean ± standard deviation.

Comparisons were performed using the Student’s *t*-test or Mann–Whitney *U* test, as appropriate.

WHF, worsening heart failure; LV, left ventricle; RV, right ventricle; GLS, global longitudinal strain; GWI, global work index; GCW, global constructive work; GWW, global wasted work; GWE, global work efficiency; LASr, left atrial reservoir strain; LAVI, left atrial volume index; TAPSE, tricuspid annular plane systolic excursion; sPAP, systolic pulmonary artery pressure.

^*^ *P* < 0.01 and **P < 0.001 compared with patients without worsening heart failure within the same heart failure category.

**Table 5:** Clinical biomarker and echocardiographic characteristics of patients with and without worsening heart failure irrespective of ejection fraction

Variable	No WHF	WHF	*P* value
*n*	142	73	
NT-pro BNP (pg/mL)	856 ± 102	1958 ± 378	<0.001
E/e′avg	11.9 ± 2.6	18.0 ± 3.8	<0.01
LAVI (mL/m^2^)	33.5 ± 8.1	35.2 ± 7.3	<0.05
LASr (%)	25.3 ± 7.3	19.6 ± 6.1	<0.01
TR PGmax (mmHg)	38.2 ± 10.2	45.3 ± 11.4	<0.02
TAPSE/sPAP	0.60 ± 0.2	0.51 ± 0.2	<0.02
RV GS (%)	20.3 ± 3.8	18.1 ± 3.2	<0.01
RV GWI (mmHg%)	762 ± 84	810 ± 92	<0.01
RV GCW (mmHg%)	648 ± 52	607 ± 48	<0.001
RV GWW (%)	110 ± 23	138 ± 32	<0.01
RV GWE (mmHg%)	86.0	81.0	<0.01

Values are presented as mean ± standard deviation.

Comparisons were performed using the Student’s *t*-test or Mann–Whitney *U* test, as appropriate.

WHF, worsening heart failure; LV, left ventricle; RV, right ventricle; GLS, global longitudinal strain; GWI, global work index; GCW, global constructive work; GWW, global wasted work; GWE, global work efficiency; LASr, left atrial reservoir strain; LAVI, left atrial volume index; TAPSE, tricuspid annular plane systolic excursion; sPAP, systolic pulmonary artery pressure; NS, non-significant.

In secondary analyses, RVGCW remained significantly associated with both subclinical WHF and HF hospitalization when analysed separately, with consistent effect direction and magnitude across analyses.

There was no association of WHF incidents with EF. Patients with WHF had more elevated filling pressures, significantly elevated sPAP, and less RV GCW compared with their counterparts within the same HF category group as shown in *Table 4*.

*Table 5* shows comparison of variables of patients irrespective of EF with WHF at the time of its detection and without WHF.

In the analysis of baseline predictors of subsequent WHF (*Table 6*), lower LASr, higher NT-proBNP, reduced TAPSE/sPAP, reduced RV longitudinal strain, and lower RVGCW were all associated with increased risk.

**Table 6:** Comparison of baseline clinical, demographic, and echocardiographic variables of patients irrespective of heart failure category with subsequent worsening heart failure and without worsening heart failure during follow-up

Variable	No WHF	WHF	*P*
n	142	73	
NT-pro BNP (pg/mL)	982 ± 154	1174 ± 213	<0.05
LASr (%)	22.1 ± 4.3	19.6 ± 3.1	<0.05
TR PGmax (mmHg)	38.2 ± 10.2	39.6 ± 11.2	NS
TAPSE/sPAP	0.57 ± 0.15	0.51 ± 0.14	<0.05
RV GS (%)	20.3 ± 3.9	19.7 ± 4.1	<0.05
RV GWI (mmHg%)	761 ± 64	768 ± 78	NS
RV GCW (mmHg%)	655 ± 72	609 ± 68	<0.001
RV GWW (%)	117	120	<0.01
RV GWE (mmHg%)	84.8	83.6	NS

Values are presented as mean ± standard deviation.

Comparisons were performed using the Student’s *t*-test or Mann–Whitney *U* test, as appropriate.

WHF, worsening heart failure; LV, left ventricle; RV, right ventricle; GLS, global longitudinal strain; GWI, global work index; GCW, global constructive work; GWW, global wasted work; GWE, global work efficiency; LASr, left atrial reservoir strain; LAVI, left atrial volume index; TAPSE, tricuspid annular plane systolic excursion; sPAP, systolic pulmonary artery pressure; NS, non-significant.

In multivariable Cox models (*Table 7*), RVGCW emerged as the strongest independent predictor of WHF. In the baseline model incorporating LV GLS, LASr, NT-proBNP, and TAPSE/sPAP, the C-index was 0.835 with an Akaike information criterion (AIC) of 689.6.

**Table 7:** Independent predictors of worsening heart failure: multivariable Cox regression analysis

Variable	HR	95% CI	*P*
Total			
LV GLS (per 1% increase, absolute value)	0.96	0.92–1.00	0.031
LASr (per % increase)	0.95	0.92–0.98	0.003
NT-proBNP (per log increase)	1.42	1.11–1.82	0.006
TAPSE/sPAP	0.91	0.84–0.99	0.028
RVGCW (per unit increase)	0.98	0.97–0.99	0.014

Multivariable Cox proportional hazards model for worsening heart failure, defined as heart failure hospitalization or adjudicated subclinical worsening heart failure (total events: *n* = 87). Hazard ratios are reported with 95% confidence intervals.

CI, confidence interval; GLS, global longitudinal strain; LASr, left atrial reservoir strain; NT-proBNP, N-terminal pro-B-type natriuretic peptide; RVGCW, right ventricular global constructive work; TAPSE, tricuspid annular plane systolic excursion; sPAP, systolic pulmonary artery pressure.

**Figure 1:**
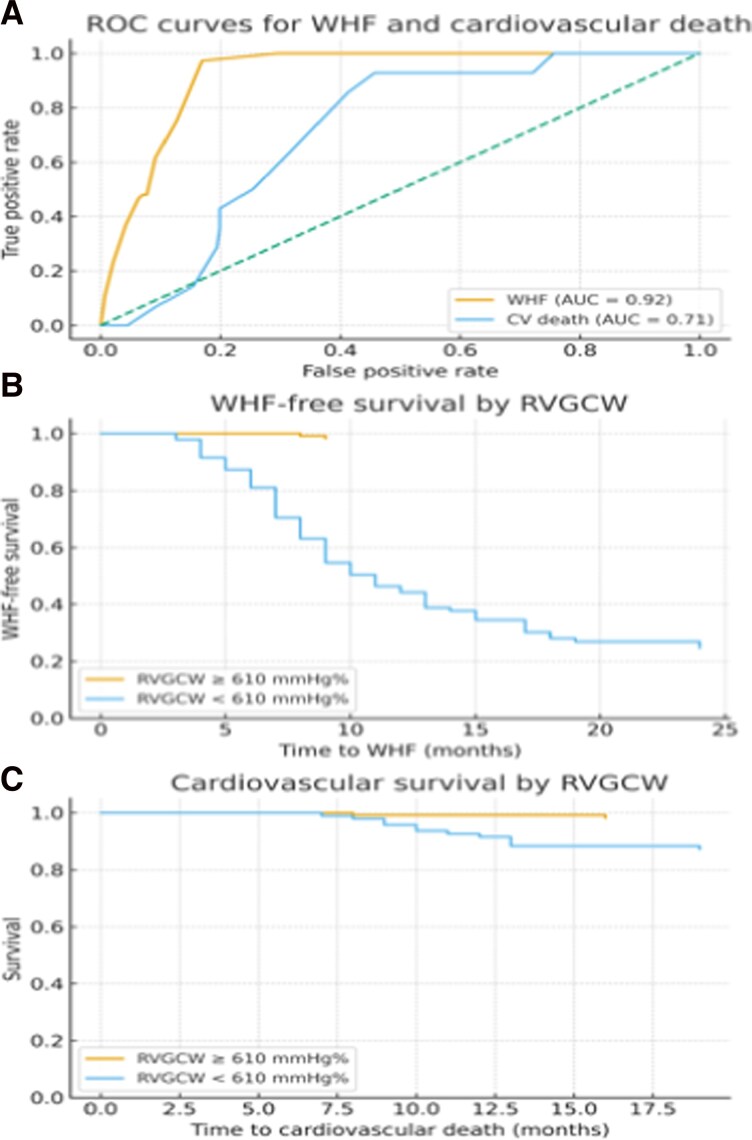
Prognostic performance of right ventricular global constructive work. (*A*) Receiver operating characteristic curves demonstrating discrimination of right ventricular global constructive work for worsening heart failure and cardiovascular mortality. (*B*) Kaplan–Meier curves for worsening heart failure-free survival stratified by right ventricular global constructive work. (*C*) Kaplan–Meier curves for cardiovascular survival stratified by right ventricular global constructive work. The right ventricular global constructive work threshold used for stratification was derived from the present cohort and is shown for illustrative purposes only; it should be regarded as hypothesis generating rather than a definitive clinical cut-off. RVGCW, right ventricular global constructive work; WHF, worsening heart failure; AUC, area under the curve. All survival times are expressed in months.

Adding RVGCW significantly improved prognostic performance, yielding a C-index of 0.891 and an AIC of 623.6. Right ventricular global constructive work provided a substantial incremental contribution (likelihood ratio χ² = 68.08, *P* < 0.0001). Sensitivity analyses using reduced models confirmed RVGCW as a robust independent predictor of WHF, irrespective of inclusion or exclusion of other correlated RV indices. In a sensitivity analysis excluding patients with chronic obstructive pulmonary disease, RVGCW remained independently associated with WHF, indicating that the observed association was not driven by pulmonary comorbidity. The prognostic performance of RVGCW for WHF and cardiovascular mortality is illustrated in *Figure 1*.

### Cardiovascular mortality

Fourteen cardiovascular deaths occurred during follow-up. In exploratory analyses, lower RVGCW was associated with increased cardiovascular mortality (HR 0.91, 95% CI 0.84–0.99, *P* = 0.020); however, due to the limited number of events (*n* = 14), these findings should be interpreted cautiously and considered hypothesis generating.

Consistent with WHF analyses, patients with lower RVGCW and impaired RV–pulmonary coupling (TAPSE/sPAP) demonstrated poorer survival (*Figure 1C*).

Although addition of RVGCW improved model discrimination metrics, interpretation of multivariable mortality analyses is limited by the low number of events.

### Receiver operating characteristic analysis

Receiver operating characteristic analysis demonstrated excellent discrimination of RVGCW for WHF (AUC = 0.92) and moderate discrimination for cardiovascular mortality (AUC = 0.71; *Figure 1A*).

Kaplan–Meier curves illustrated significantly reduced WHF-free and cardiovascular survival among patients with RVGCW values below a data-derived cohort-specific threshold (*Figure 1B* and *C*). This threshold was derived from the present cohort and is shown for illustrative purposes only; it should be regarded as hypothesis generating rather than a definitive clinical cut-off.

## Discussion

In this prospective study of ambulatory patients with chronic HF spanning the full spectrum of ejection fraction, we demonstrate that RVGCW, an echocardiographic pressure–strain–integrated marker of RV performance, is a powerful and independent predictor of both WHF and cardiovascular mortality. Right ventricular global constructive work provided substantial incremental prognostic value beyond established indices of left-sided systolic function, atrial function, and RV–arterial coupling. These findings highlight the central role of RV myocardial mechanics in HF progression and suggest that RVGCW can identify physiologically vulnerable patients well before clinical decompensation becomes evident.

An important observation is that although HFrEF, HFmrEF, and HFpEF exhibit distinct LV structural and functional phenotypes, the trajectory towards WHF did not mirror these phenotypic differences. This reinforces the concept that LV systolic indices—including advanced measures such as GLS and LVMW—are not the principal drivers of early HF destabilization. Left ventricular filling pressures were significantly elevated at the time WHF was detected but did not differ at baseline, indicating that congestion-related parameters have strong diagnostic but limited predictive value.

By contrast, the RV phenotype appears less tied to HF category and more reflective of right-sided haemodynamic burden transmitted from the left heart. Because pulmonary pressures may rise irrespective of LVEF, RV constructive work integrates both intrinsic RV contractility and afterload, thereby capturing a more universal and load-adjusted expression of HF advancement. This mechanistic profile is consistent with our finding that reductions in RVGCW precede overt WHF. The conceptual framework linking progressive pulmonary vascular load, compensatory LV work, loss of energetic efficiency, and transition to WHF is summarized in the *Graphical abstract*.

Traditional markers—NT-proBNP, LASr, E/e′, and TAPSE/sPAP—were each individually associated with WHF; however, only RVGCW consistently retained independent prognostic significance in multivariable analysis and provided the greatest incremental improvement in model discrimination. Because HRs are expressed per unit increase, direct comparison of HR magnitudes across variables with different scales (e.g. RVGCW, NT-proBNP, and LASr) is not appropriate; instead, independent significance and incremental improvement in model performance more accurately reflect comparative prognostic strength.

### Right ventricular global constructive work as an integrated marker of right ventricular energetic efficiency

The prognostic value of RVGCW should not be interpreted solely as a marker of early RV–pulmonary arterial uncoupling. Although RV strain and TAPSE/sPAP were also impaired at the time of WHF detection, these indices primarily reflect deformation or coupling, whereas RVGCW integrates myocardial deformation with afterload, providing an energetic perspective on RV performance.

The observed increase in RVGWI at WHF onset likely reflects a compensatory increase in total RV work in response to rising pulmonary pressures. However, this increase occurs at the expense of efficiency, as evidenced by a disproportionate reduction in constructive work and an increase in wasted work.

Thus, RVGCW captures the inability of the RV to translate increased workload into effective forward work, explaining why it remains independently prognostic when other RV metrics lose significance.

### Comparison with previous studies

Previous investigations of RVMW have largely focused on feasibility or cross-sectional associations with invasive haemodynamics in pulmonary hypertension or valvular disease.^20,22–26^ These proof-of-concept studies demonstrated the technical feasibility of RVMW and its association with RV dysfunction, but did not evaluate its prognostic relevance in a broad chronic HF population.

In contrast, our study extends this work by demonstrating that RVGCW predicts longitudinal clinical deterioration in ambulatory chronic HF patients and provides incremental prognostic value beyond conventional echocardiographic and biomarker-based markers. To our knowledge, this is the first study to show a robust, independent association between RVGCW and WHF across HF phenotypes.

Notably, the magnitude of association observed for RVGCW exceeded that typically reported for LVMW or GLS, emphasizing the sensitivity of RV work to subtle changes in pulmonary haemodynamics and HF progression.

### Mortality signal

Fourteen cardiovascular deaths occurred during follow-up. Given the limited number of events, mortality analyses should be considered exploratory. Although lower RVGCW was associated with reduced survival, these findings should be interpreted cautiously and viewed as hypothesis generating rather than definitive and require confirmation in larger cohorts.

### Clinical implications

Our findings carry several important clinical implications:

Early risk stratification:
  RVGCW may help identify patients at high risk for decompensation during periods of apparent stability, supporting closer follow-up, earlier intensification of diuretics, or optimization of GDMT.
Better characterization of HF phenotypes:
  Prognostic value was independent of EF, indicating that RVGCW reflects HF severity across HFrEF, HFmrEF, and HFpEF, where conventional markers often underperform.
Potential for monitoring treatment response:
  Because RVGCW integrates both contractility and afterload, it may serve as a dynamic marker to monitor response to therapies targeting pulmonary pressures, congestion, or RV function.
4. Integration into multiparametric evaluation:
  Given its incremental value over NT-proBNP, LA strain, and TAPSE/sPAP, RVGCW could enrich multiparametric prediction models and guide more personalized care.

### Limitations

This study has several important limitations. First, the cohort was highly selected: all patients were in sinus rhythm, relatively young, and receiving optimized GDMT and had adequate acoustic windows with analysable TR envelopes. As a result, external validity is limited, and findings may not be generalizable to older HF populations, patients with atrial fibrillation, advanced comorbidity burden, or suboptimal echocardiographic windows.

Second, although WHF adjudication followed a structured process, definitions of subclinical WHF are not yet universally standardized and may vary across centres.

Third, the number of cardiovascular deaths was modest, limiting the power of mortality analyses. Finally, RVMW remains a relatively novel parameter requiring dedicated software and further multicentre validation.

## Conclusions

In ambulatory patients with chronic HF and preserved sinus rhythm, RVGCW emerged as a robust echocardiographic marker of WHF, providing independent and incremental prognostic information beyond conventional echocardiographic parameters and circulating biomarkers. Right ventricular global constructive work integrates right ventricular deformation, afterload, and energetic efficiency, thereby capturing early maladaptive RV–pulmonary interactions preceding overt clinical deterioration. Importantly, the present findings apply to a selected population with adequate image quality and should be interpreted within this context. While the identified RVGCW threshold enabled meaningful risk stratification within this cohort, it should be regarded as hypothesis generating rather than as a definitive clinical cut-off. Multicentre, multi-vendor validation studies across broader HF populations are required before RVGCW can be considered for routine clinical implementation.

### Highlights/what’s new?

1. Right ventricular global constructive work is a strong, independent predictor of worsening HF and mortality across EF phenotypes.
2. Right ventricular global constructive work provides incremental prognostic value beyond NT-proBNP, LV GLS, LASr, LA volume, and TAPSE/sPAP.
3. Right ventricular myocardial work detects vulnerability before clinical decompensation, whereas LV parameters did not predict WHF.
4. Right ventricular myocardial work < 610 mmHg% identifies high-risk patients during compensated phases.

### Clinical implications

1. Right ventricular myocardial work may support earlier identification of patients at risk for HF destabilization.
2. Right ventricular myocardial work can be integrated into multiparametric risk models in ambulatory HF.
3. Could inform follow-up intervals, GDMT optimization, and targeted monitoring strategies.

## Supplementary Material

oeag019_Supplementary_Data

## Data Availability

The data underlying this article will be shared on reasonable request to the corresponding author.
