# Supplementary figures and images for: Right ventricular global constructive work as an echocardiographic predictor of worsening heart failure
Source: Eur Heart J Open. 2026 Feb 17;6(1):oeag019. doi: 10.1093/ehjopen/oeag019 (PMC12958019; doi:10.1093/ehjopen/oeag019)

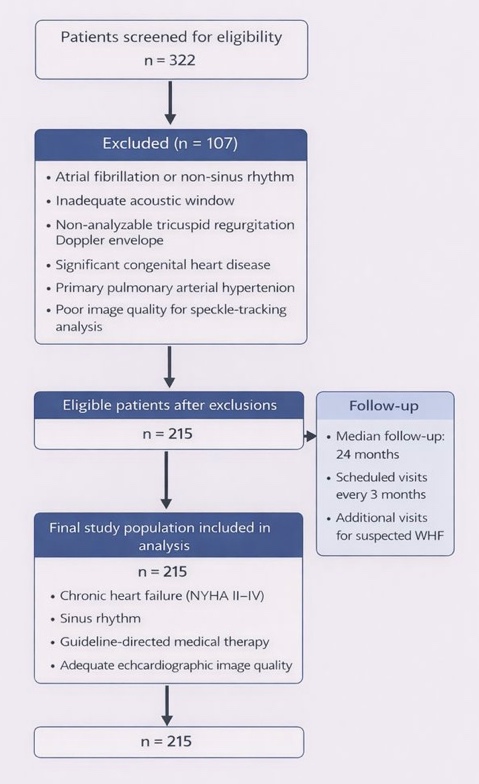


Figure S1

Supplement: oeag019_Supplementary_Data [file oeag019_supplementary_data.zip › Figure S1.docx]

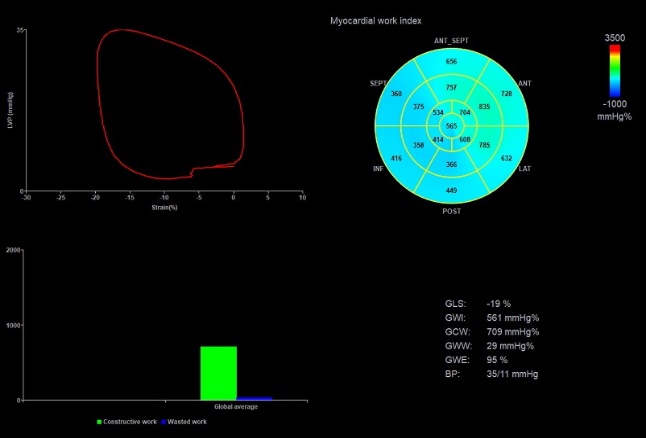


Figure S2

Supplement: oeag019_Supplementary_Data [file oeag019_supplementary_data.zip › Figure S2.docx]
